# Supplementary material for: Iterating toward change: Improving student-centered teaching through the STEM faculty institute (STEMFI)
Source: PLoS One. 2023 Aug 17;18(8):e0289464. doi: 10.1371/journal.pone.0289464 (PMC10434963; doi:10.1371/journal.pone.0289464)
Supplement: S1 File — (DOCX) [file pone.0289464.s001.docx]

Supplementary Materials

**Interview Protocol**

**STEMFI feedback**

1. What is your overall feedback about the STEMFI experience?
2. What was most motivating for you to engage in the STEMFI activities? ($600, workshop, mentoring, cohort meetings, items for your portfolio, etc.)
3. Which STEMFI activities were most helpful? (summer workshop, mentoring, cohort meetings, stipend, etc.)
   1. How were they helpful?
4. Which STEMFI activities were least helpful? (summer workshop, mentoring, cohort meetings, stipend, etc.)
5. How could they be improved?
6. In particular, we would like to know how the summer workshop functioned. What was most helpful from the summer workshop? What was least helpful? How could the summer workshop be improved?
7. Any other suggestions for how we could improve STEMFI?

**Student-Centered Change**

1. Can you tell me the story about what you changed in your class this year?
   1. What new strategy did you implement?
   2. How did you choose that part of your class and that strategy?
   3. How did you develop the activity/materials?
   4. How much time do you think it took to develop this?
   5. How was it received by students?
   6. What did you like/dislike about it?
   7. How will you modify it in future years?
2. We really want to understand your experience because it will be helpful for others in the future. During this experience was there anything that surprised you? Or was an aha! Moment?
   1. Probe for supports/drivers: What made changes easier?
   2. Probe for drivers and barriers: What made the change difficult if anything?
   3. As you were making this change, what did you do to overcome your challenges and find solutions?
3. What would best help you with pedagogical changes in the future? (probe to ensure they’re talking about student-centered teaching activities)
4. Imagine your department just hired a new faculty member, and you’ve been assigned as their mentor. How would you help them use student-centered teaching strategies?

**Theory of Planned Behavior (if not addressed previously)**

1. Have your attitudes about student-centered teaching changed over the course of your participation? If so, how?
2. Has your confidence to use student-centered teaching methods changed? How?
3. How do you plan to implement student-centered teaching in the future?
4. How have you dealt with issues such as:
   1. Time?
   2. Lack of resources?
   3. Classroom setup?
5. How have the following responded to the changes you’ve made:
   1. Students
   2. Colleagues (on campus or in professional organizations)
   3. Department chair or dean
