# Supplementary material for: Iterating toward change: Improving student-centered teaching through the STEM faculty institute (STEMFI)
Source: PLoS One. 2023 Aug 17;18(8):e0289464. doi: 10.1371/journal.pone.0289464 (PMC10434963; doi:10.1371/journal.pone.0289464)
Supplement: Attachment — Submitted filename: PLOS ONE FEEDBACK.pdf [file pone.0289464.s004.pdf]

Overall, my biggest feedback is what Reviewer 1 previously mentioned about the post-observation not being at random and only occurring the single time when the pre-observation occurred multiple times. This is problematic as the instructor could be performative in their methods and not actually adopting SCT strategies. Additionally, only doing the post-observation once, is a similar issue that leads for the data to not be as generalizable on if the class was a one-off or if the instructor has really adopted SCT strategies. Overall, I think to help remediate this, the categorization of faculty needs to be written differently as you can't confirm that they are now student-centered instructors, but rather that they can use the techniques efficiently. This needs to be made clear in the manuscript.
