# Supplementary material for: Iterating toward change: Improving student-centered teaching through the STEM faculty institute (STEMFI)
Source: PLoS One. 2023 Aug 17;18(8):e0289464. doi: 10.1371/journal.pone.0289464 (PMC10434963; doi:10.1371/journal.pone.0289464)
Supplement: Attachment — Submitted filename: Review PONE.docx [file pone.0289464.s002.docx]

Review PONE-D-22-33194

Feb 2, 2023

Iterating toward change: improving student-centered teaching through the STEM

faculty institute (STEMFI)

There is ample evidence to support the claim that active learning improves learning for undergraduate STEM students. More recently these teaching practices have also been shown to decrease the equity gap in STEM classrooms. The challenge academia faces is how to get more faculty to implement these transformative and equity producing teaching practices. This paper presents an assessment of one faculty development program (STEMFI) implemented at an R1 university.

The authors have used a strong theoretical foundation, Ajzen’s Theory of Panned Behavior, as both a basis of the design of their program and a framework for assessing the effectiveness of STEMFI. The authors have used both a quantitative and qualitative approach as assessment tools which could provide more insight on the process of change for future professional development programs.

**Major Concerns**

#1. Including COPUS as an objective empirical assessment of participants actual teaching practices in the classroom had the potential of providing a rigorous assessment the program. However, the fact that the researcher allowed the STEMFI participants to select the classes to be COPUSed at the end of the program rather than randomly selecting classes as they did in the first semester, severely undermines the rigor of the results. By allowing the faculty to select the post-classses to COPUS, they introduced considerable bias into their results. Given that this study is now concluded, I am not sure how to resolve this issue.

#2. Combining both a quantitative and qualitative analysis was a nice idea. However, I did not see enough differentiation among the qualitative results for each categorization of faculty post involvement in the program to be helpful. It felt like all reform groups said the same thing yet the authors interpreted the results differently and went beyond the scope of the coding rubric they established and presented in the paper.

\# 3. Under the Explanatory results-Descriptive profiles section of the paper you left out two key categories of participants: Interactive Reformers and Didactic Non-reformers. Neither of these groups changed over the course of the program. In many ways your qualitative data from these two groups would be as important or more important than the data from the groups who did change.

**Minor Concerns**

**Introduction**

On line 43 the authors introduce the term student-centered teaching (STC). Could they please explain why they are using this term rather than active learning or evidence-based teaching practices. Using multiple terms for the same teaching methods can introduce confusion to the professional development field of study.

Line 60. As the authors only cite one study [4], it would be best if this sentence began with the qualifier “some”. Also it is not clear what you mean by “including those with pedagogical training”. What does “those” refer to.

Ln 63-67. A key citation that is missing from this section is “Henderson, C., Beach, A., & Finkelstein, N. (2011). Facilitating change in undergraduate STEM instructional practices: an analytic review of the literature. *Journal of Research in Science Teaching*, 48(8), 952–984. https:// doi. org/ 10.1002/ tea. 20439” Henderson et al. review of 191 articles describing professional development, very succinctly concludes that are two common practices that do not work and he identifies four practices of successful programs. STEMFI includes all of these.

Ln 70. An additional reference by Ann Austin would be most appropriate here as well.
Austin, A. E. (2011). Promoting evidence-based change in undergraduate science education. In Fourth committee meeting on status, contributions, and future directions of discipline-based education research.

There is a good deal of repeated material in the Introduction and Methods section of the paper. I realize that the authors are try to align different components of STEMFI with their theoretical framework but it gets confusing. For instance, the information on Ln 77-81 is repeated in Methods and really does not fit in the introduction.

This issue arises again in Ln 115-128. Most of this content in repeated in the Methods section starting at LN 149. Ln 125-128 particularly is methods rather than introduction.

Possibly the authors could move and consolidate the description “STEMFI Program” to the Introduction and address how you collected COPUS data and coded interview data in Methods.

**Methods**

Please present aggregate information on the gender and ethnicity of the participants.

Please state how the four random class session were selected. Was COPUS conducted in person or by watching a video of class. If in person, was the faculty member aware of that this class was being observed? For the Post-COPUS observation, was this done at the end of the second term that they taught the same course or at the end of the first term they taught the course?

How long was the summer workshop and were faculty financially compensated for attendance.

Please indicate who served as peer mentors and how were mentors paired with determined.

One major concern is your post-COPUS observation method. You state that you allowed the faculty to select the post-course to be observed. In the paper you gave your explanation for this (ln 185-187). However, I see this as a major limitation of your study. It is possible that the faculty member only used the new teaching method on those 3-4 days and used didactic teaching methods on the other 40 days of teaching. Therefore, did they really change and was your categorization of them (fig 2) correct. The post-COPUS score would indicate how well they implemented the select group or type of evidence-based teaching they used that day but may not reflect their actual teaching method. You need to put many more qualifiers on this part of your results.

The categories you created to classify participants are very clever and the figure 2 is a nice representation of that categorization method.

**Interview Protocol**

You state that your interview protocol was to align with the Theory of Planned Behavior (TPB) and address attitudes, student response and confidence. However, you then created four themes but there were only three attributes of the TPB. Two of your themes 1) attitude and 2 student responses align directly with TPB but motivation and challenges do not align. This was confusing. Could you explain how the TPB informed the last two themes of motivation and challenges. You may have to do this in results section.

The sentences from 224 to 227 do not fit under Interview Protocol but rather should be a stand alone paragraph indicating how you will triangulate your quantitative and qualitative data.

**Results**

**Quantitative**

Please add the academic rank to the participants in each category. You could add three rows under your existing row at bottom of Fig 2 and indicate the number of faculty who are Assist., Assoc., Full Professors. This would help the reader see if newer faculty are more likely to change than established faculty.

**Qualitative results**

The sentence on Ln 249 starting with “Attitudes… should be the beginning of a new paragraph as you are now presenting results on your first theme of attitudes.

The quotes you presented nicely support the binary categories you established.

**Explanatory Results- Descriptive profiles**

This section was challenging to follow. I found myself creating a chart with the four themes in the first column and each column was one of your faculty categories, i.e. Beginning Reformer, Dramatic Reformer, etc. and putting the binary classification in each appropriate cell. I strongly suggest you create such a table.

Also, rather than just presenting the results from the coding of the interviews there is a good deal of interpretation of these results. Keeping the interpretation of results for the discussion section could be helpful to the reader and allow more compare and contrasting between faculty categories.

I am also concerned that the results for this section do not add further clarity to your results. When I look at the results for motivation all four faculty categories show intrinsic motivation and all four show logistical challenges. On Ln 430, you indicate that student centered reformers *perceived* the challenge differently but what method did you use to code *perception*? The authors may be reading too much into faculty answers.

When you presented results for Advanced Reformer as well as Student-centered Reformer, you changed the order of presentation of the four themes. Please keep the same order as the others, i.e. attitude, student response, motivation and then challenges. This section has high cognitive load for the reader and keeping the same order of presentation of themes within each category would help decrease the load.

I was very much looking forward to your qualitative triangulation of the Interactive and Didactic Reformer. What did you find in your interviews with them that could shed light on faculty who do not change. These are really the major challenge for future professional development programs. It is necessary that you provide the explanatory results for these two profiles.

**Discussion**

As I mentioned earlier, the interpretation of result found in the results section would fit better in the discussion section.

I did not see data presented to support your claim in Ln 455 “By addressing the attitudes….”

Ln. 471. The sentence starting with “our results…” needs a qualifier as not all faculty changed categories by the end of the program. I would suggest “SCT was instrumental in aiding *80% (or the majority of participants)* STEMFI participants.

Ln 473. I am not clear how STEMFI program specifically addressed the participants attitudes.
