# Supplementary material for: Iterating toward change: Improving student-centered teaching through the STEM faculty institute (STEMFI)
Source: PLoS One. 2023 Aug 17;18(8):e0289464. doi: 10.1371/journal.pone.0289464 (PMC10434963; doi:10.1371/journal.pone.0289464)
Supplement: Attachment — Submitted filename: Response to Reviewers.docx [file pone.0289464.s003.docx]

Response to Reviewers:

## Reviewer #1:

## I have attached a word document with this information. I have two main concerns with this paper. 1. The classes selected to observe with COPUS at the end of the program were not randomly selected but rather the participants selected the courses to be observed. This introduces consider bias into the study design and undermines the results presented. 2. The results from the qualitative analysis did not further differentiate the participants in each of the categories the researchers presented and at times different arguments were presented for the same findings.

**Major Concerns**

#1. Including COPUS as an objective empirical assessment of participants actual teaching practices in the classroom had the potential of providing a rigorous assessment the program. However, the fact that the researcher allowed the STEMFI participants to select the classes to be COPUSed at the end of the program rather than randomly selecting classes as they did in the first semester, severely undermines the rigor of the results. By allowing the faculty to select the post-classses to COPUS, they introduced considerable bias into their results. Given that this study is now concluded, I am not sure how to resolve this issue.

Thank you for this feedback. We agree that allowing participants to choose the class post-workshop would be biased if we were trying to show that the instructor was truly reformed in all aspects of their teaching. However, we were just trying to show that instructors could take what they had learned and successfully implement it in their courses (hence, we wanted to see those particular courses). Given this distinction, however, we agree that our language throughout was perhaps not so clear on this point. We have gone through the paper and cleaned that up. We have also made a note of this in our limitations section.

#2. Combining both a quantitative and qualitative analysis was a nice idea. However, I did not see enough differentiation among the qualitative results for each categorization of faculty post involvement in the program to be helpful. It felt like all reform groups said the same thing yet the authors interpreted the results differently and went beyond the scope of the coding rubric they established and presented in the paper.

This is an interesting perspective and it made us re-think how we described our categories. We agree that it was difficult to differentiate among the reformer types with the qualitative data and we decided that perhaps that was not the point. We have gone in and rewritten the qualitative section to be more about the four themes that emerged in conjunction with the TPB framework, with suggestions for trends that match the profiles. But, we emphasize that it is not always a direct relationship between them (i.e., there is a lot of overlap). We have also included descriptions of our didactic non-reformers.

\# 3. Under the Explanatory results-Descriptive profiles section of the paper you left out two key categories of participants: Interactive Reformers and Didactic Non-reformers. Neither of these groups changed over the course of the program. In many ways your qualitative data from these two groups would be as important or more important than the data from the groups who did change.

See above - we have included additional descriptions of non-reformers.

**Minor Concerns**

**Introduction**

On line 43 the authors introduce the term student-centered teaching (STC). Could they please explain why they are using this term rather than active learning or evidence-based teaching practices. Using multiple terms for the same teaching methods can introduce confusion to the professional development field of study.

We prefer the term “Student-Centered Teaching” to emphasize the constructivist approach that we were teaching. Active learning seems too broad (i.e., teaching can be active and not constructivist). Evidence-based practices is also just not as descriptive. We have made sure we consistently use that term throughout.

Line 60. As the authors only cite one study [4], it would be best if this sentence began with the qualifier “some”. Also it is not clear what you mean by “including those with pedagogical training”. What does “those” refer to.

Thank you for catching this. We have changed this sentence.

Ln 63-67. A key citation that is missing from this section is “Henderson, C., Beach, A., & Finkelstein, N. (2011). Facilitating change in undergraduate STEM instructional practices: an analytic review of the literature. *Journal of Research in Science Teaching*, 48(8), 952–984. https:// doi. org/ 10.1002/ tea. 20439” Henderson et al. review of 191 articles describing professional development, very succinctly concludes that are two common practices that do not work and he identifies four practices of successful programs. STEMFI includes all of these.

Thank you for the excellent paper and suggestion. We have added this.

Ln 70. An additional reference by Ann Austin would be most appropriate here as well.

Austin, A. E. (2011). Promoting evidence-based change in undergraduate science education. In Fourth committee meeting on status, contributions, and future directions of discipline-based education research.

Another useful citation. Thank you..

There is a good deal of repeated material in the Introduction and Methods section of the paper. I realize that the authors are try to align different components of STEMFI with their theoretical framework but it gets confusing. For instance, the information on Ln 77-81 is repeated in Methods and really does not fit in the introduction.

We have removed that paragraph.

This issue arises again in Ln 115-128. Most of this content in repeated in the Methods section starting at LN 149. Ln 125-128 particularly is methods rather than introduction.

Possibly the authors could move and consolidate the description “STEMFI Program” to the Introduction and address how you collected COPUS data and coded interview data in Methods.

We have removed redundancy in the Introduction and preferred to keep all methodologies in the methods.

**Methods**

Please present aggregate information on the gender and ethnicity of the participants.

We have included gender. Ethnicity data was not collected.

Please state how the four random class sessions were selected. Was COPUS conducted in person or by watching a video of class. If in person, was the faculty member aware of that this class was being observed? For the Post-COPUS observation, was this done at the end of the second term that they taught the same course or at the end of the first term they taught the course?

We have further clarified this in the methods.

How long was the summer workshop and were faculty financially compensated for attendance.

We have added this to the methods.

Please indicate who served as peer mentors and how were mentors paired with determined.

We have added this, as well

One major concern is your post-COPUS observation method. You state that you allowed the faculty to select the post-course to be observed. In the paper you gave your explanation for this (ln 185-187). However, I see this as a major limitation of your study. It is possible that the faculty member only used the new teaching method on those 3-4 days and used didactic teaching methods on the other 40 days of teaching. Therefore, did they really change and was your categorization of them (fig 2) correct. The post-COPUS score would indicate how well they implemented the select group or type of evidence-based teaching they used that day but may not reflect their actual teaching method. You need to put many more qualifiers on this part of your results.

Thank you. We have commented on this above and we have made additions to our paper to indicate this potential limitation.

The categories you created to classify participants are very clever and the figure 2 is a nice representation of that categorization method.

Thank you!

**Interview Protocol**

You state that your interview protocol was to align with the Theory of Planned Behavior (TPB) and address attitudes, student response and confidence. However, you then created four themes but there were only three attributes of the TPB. Two of your themes 1) attitude and 2 student responses align directly with TPB but motivation and challenges do not align. This was confusing. Could you explain how the TPB informed the last two themes of motivation and challenges. You may have to do this in results section.

Thank you for asking for this clarification. In our rewrite of our qualitative data results section, we have better clarified how these fit. It’s important to note that the interviews were only semi-structured *and* the data were analyzed using an emergent themes approach. Thus, two themes that naturally emerged went beyond TPB. We have included this in our methods.

The sentences from 224 to 227 do not fit under Interview Protocol but rather should be a stand alone paragraph indicating how you will triangulate your quantitative and qualitative data.

Done

**Results**

**Quantitative**

Please add the academic rank to the participants in each category. You could add three rows under your existing row at bottom of Fig 2 and indicate the number of faculty who are Assist., Assoc., Full Professors. This would help the reader see if newer faculty are more likely to change than established faculty.

We felt that adding it to the figure was just a little too messy. But we have included that information in the text of the results.

**Qualitative results**

The sentence on Ln 249 starting with “Attitudes… should be the beginning of a new paragraph as you are now presenting results on your first theme of attitudes.

Okay.

The quotes you presented nicely support the binary categories you established.

Thank you!

**Explanatory Results- Descriptive profiles**

This section was challenging to follow. I found myself creating a chart with the four themes in the first column and each column was one of your faculty categories, i.e. Beginning Reformer, Dramatic Reformer, etc. and putting the binary classification in each appropriate cell. I strongly suggest you create such a table.

Also, rather than just presenting the results from the coding of the interviews there is a good deal of interpretation of these results. Keeping the interpretation of results for the discussion section could be helpful to the reader and allow more compare and contrasting between faculty categories.

I am also concerned that the results for this section do not add further clarity to your results. When I look at the results for motivation all four faculty categories show intrinsic motivation and all four show logistical challenges. On Ln 430, you indicate that student centered reformers *perceived* the challenge differently but what method did you use to code *perception*? The authors may be reading too much into faculty answers.

When you presented results for Advanced Reformer as well as Student-centered Reformer, you changed the order of presentation of the four themes. Please keep the same order as the others, i.e. attitude, student response, motivation and then challenges. This section has high cognitive load for the reader and keeping the same order of presentation of themes within each category would help decrease the load.

I was very much looking forward to your qualitative triangulation of the Interactive and Didactic Reformer. What did you find in your interviews with them that could shed light on faculty who do not change. These are really the major challenge for future professional development programs. It is necessary that you provide the explanatory results for these two profiles.

We agree with you completely. We have significantly changed this section to try to make it clearer and more useful for interpretation. We have also included descriptions for non-reformers.

**Discussion**

As I mentioned earlier, the interpretation of result found in the results section would fit better in the discussion section.

Agreed. We have tried to move our interpretations all to the discussion.

I did not see data presented to support your claim in Ln 455 “By addressing the attitudes….”

We have modified this sentence to be more clear.

Ln. 471. The sentence starting with “our results…” needs a qualifier as not all faculty changed categories by the end of the program. I would suggest “SCT was instrumental in aiding *80% (or the majority of participants)* STEMFI participants.

We have modified this sentence.

Ln 473. I am not clear how STEMFI program specifically addressed the participants attitudes.

We have clarified the claim in this paragraph.

Reviewer #2: Overall, this is a much needed study in the field. However, there are some issues with the qualitative data that need to be addressed. The most major issue is that the qualitative data needs to revised to make it much more clear of the themes and the process of finding these themes. For example: how did the themes emerge? How prevalent are these themes? How were the themes found (i.e., using inductive or deductive approaches). Commentary and explanation of the provided quotes are also necessary in order to explain how the quotes are related to the theme. Additionally, the triangulation between the COPUS and interview data needs to be significantly more explained than it is now. Some visuals may be useful to represent the qualitative data.

Thank you for the feedback. We have added a little bit more detail to methods explaining the thematic analysis. We did not, however, quantify themes as that was not the focus of the analysis. We do agree that the profiles portion of our quantitative analysis (i.e., the triangulation between copus “reformer types” and interview data) was not as clear as we had hoped (as another reviewer pointed out). We have opted to focus on the four themes and their relation to the Theory of Planned Behavior. And we have explained in our discussion how the qualitative data was not able to differentiate “reformer types” like the quantitative could.

Here is some line feedback I have as well:
Line 53: Often workshops are one-off professional development opportunities. You seem to be describing a course redesign institute here. Being clear about what you mean by workshops will be important.

This was a good point. We have added additional detail to clearly show that this program is much more involved than a one-off PD experience.

Line 85: How framework is connected to teaching and learning is important to include.

Great point. We have added additional detail to that paragraph.

Line 96-98: Additional explanation of how your description of the program is related to the attitude toward the behavior is important here.

Thank you. We have clarified.

Reviewer #3: The authors of this manuscript used Ajzen’s Theory of Planned Behavior to design the STEM Faculty Institute (STEMFI) and categorize the types of reformers who completed this professional development program. Pre and post surveys, interviews and classroom observations were used to create descriptive profiles of participants who changed their instructional styles following STEMFI. Based on the improvements of the majority of the participants, the researchers asserted that the Theory of Planned Behavior was an effective framework for producing change in faculty behavior.

Overall, I think that this manuscript was well-written with a sound rationale and sophisticated design, and analysis. I have no request for modifications. I felt inspired while reading this manuscript and I think that this content will be a substantial contribution to the study of faculty professional development programs.

Thank you so much!
