# Supplementary material for: Iterating toward change: Improving student-centered teaching through the STEM faculty institute (STEMFI)
Source: PLoS One. 2023 Aug 17;18(8):e0289464. doi: 10.1371/journal.pone.0289464 (PMC10434963; doi:10.1371/journal.pone.0289464)
Supplement: Attachment — Submitted filename: ReReview PLoS One PONE-D-22-33194.docx [file pone.0289464.s005.docx]

May 6, 2023

Re-Review PLoS One PONE-D-22-33194

The authors have done a very nice job of revising their manuscript based on my initial comments. I find the manuscript is now easier to read and presents a stronger case for their findings. I have only a few minor changes and I do not need to rereview these minor changes.

Unfortunately the manuscript did not come with line numbers so I will only be able to use page number to indicate areas for change.

**Page 7**

You start off this section indicating that the program consisted of three phases: pre-, during, and post-workshop. You indicate the Pre-workshop content and in paragraph 2 on page 8 you identify phase three. Where is Phase 2. It would be nice if paragraph one on page 8 began with the phrase, Phase 2 involved faculty participation in a on-time workshop.

On the other hand you could just drop the use of the term “phases:.

**Page 9 top**

“observed using COPUS to measure their post teaching behaviors. Participants chose which classes would be observed.”

Please remove the sentence “Participants chose which classes would be observed” as you more fully explain this in the next section about Observations. This much detail feels out of place here.

**Page 9 bottom**

“Post-observations were made in the first semester they taught following their participation and were requested by the participant in order to showcase the new techniques they were planning to use.”

This is a nice sentence that addresses the non-random selection of post-workshop courses to COPUS.

The word “selected” would be a better word than “requested”

**Page 10**

“likewise those who were already using student-centered strategies and continued

being student**-**centered were labeled “Student**-**Centered Reformers”.

I found it odd that a faculty member that started as student-Centered and remained at student -centered would be titles a “reformer” as in fact they did not reform they just kept doing what they were already doing. If however they “broadened their strategies’ as was stated for the interactive to interactive faculty than I could accept labeling them as reformers. Please add more text here to show how they can be considered “reformers”.

This will also impact your statement on page 12 that states 35 of 41 participants changed their teaching enough to be “reformers”. If faculty who remained in their category ( Inter to Inter, or SC to SC) and did not add new strategies, they should not be termed reformers.

**Page 13-top of page**.

“so they can only hint at potential differences between reforming attitudes.”

I would rewrite as “so they can only hint at potential differences between reformers.”

**Page 13**

You italicized the term *in transition* but did not italicize the dichotomus pair “Fully Reformed”. As you do not italicize the dichotomous pairs under the other themes please remove the italics from *in transition.*

**Page 21**

Student-centered reformers were those who already had demonstrable experience implementing SCT strategies coming into this experience. Their attitudes can teach us several

things. First, we see fully reformed attitudes among this group

The middle sentence, Their attitudes.. needs a rewrite. I suggest the following

Student-centered reformers were those who already had demonstrable experience implementing SCT strategies coming into this experience. We found that this groups has fully reformed attitudes.

**Page 21- bottom**

Non-reformers displayed many of the same attitudes as those who chose to make

measurable changes. However, we can learn a few things from them that can help inform future efforts.

The second sentence felt a bit too casual with your reader. I would just remove it.

**Page 24 Conclusions**

“According to the established literature, students who learn through a more student-centered

approach have a greater overall retention of information [e.g., 31].”

I have read citation 31 Smith et al. 2014. They conducted a survey of teaching practices across their university using COPUS and had faculty self-report the practices they use. They noted a high degree of alignment between COPUS results and self-reports. I did not see any mention of “greater overall retention of information”. Please remove this citation.

You may need to remove this whole last sentence because I am unaware of any established literature that has shown greater overall retention of information. There are many citations that support increased exam scores but none that I know of that deal with retention.
