# Supplementary material for: Iterating toward change: Improving student-centered teaching through the STEM faculty institute (STEMFI)
Source: PLoS One. 2023 Aug 17;18(8):e0289464. doi: 10.1371/journal.pone.0289464 (PMC10434963; doi:10.1371/journal.pone.0289464)
Supplement: Attachment — Submitted filename: Review PONE 5-7-23v2docx.docx [file pone.0289464.s006.docx]

Review PONE D-22-33194R1

Iterating toward change: improving student-centered teaching through the STEM

faculty institute (STEMFI)

Revision 1 has improved this manuscript measurably. My comments are intended to suggest further clarification within the manuscript. I read the track-changes copy provided in PONE and line numbers do not appear -- so I used bullets to separate my comments.

• Title: “Iterating toward change” is awkward. I agree, there are three key points - time, SCT, and STEMFI. Change over time -- seems key to me. Change over time: student-centered teaching increases via STEMFI - spell out.

•The purpose of this work is now clearly articulated. “To address these barriers, the STEM Faculty Institute (STEMFI) program was created as a year-long faculty development program with a dual purpose to support lasting faculty change to SCT and to better understand what drives faculty to make that change.”

“Lasting” in the context of this study is one year. Consider using “persistence” or “persistent”.

•Rationale for TPB is justified and is a useful framework for this study.

In the section on Barriers for Instructional Change

•p4 “departmental affairs” Consider “departmental culture” - a term that is more encompassing and aligns with the literature in I/O Psych (Industrial and Organizational Psychology) e.g. Ford KJ et al 2018 Psychol. Organ. Behav. 2018.

•Classroom set-up and/or classroom design -- not really an “external factor” - rather a logistics and equipment issue that I suggest is part of the institution/department fabric rather than external.

Authors claim they are able to directly measure changes in faculty teaching and attitudes and therefore understand the effectiveness of the STEMFI program in facilitating those changes.

•The use of “understand” is problematic - what does it mean? This research “describes” the effectiveness of STEMFI.

“A goal of four class-length observations were [was] made for each

faculty participant (although some received only three observations).” Awkward sentence.

•Suggest: Faculty participants were observed 3-4 times during the study. The fourth observation was added to cohort 3 based on the recommendation for 4 observations with COPUS (Stains et al.)

“We then created dichotomous categories within each theme. Through the lens of the

Theory of Planned Behavior, we triangulated these categories with COPUS data to make loose

hypotheses about the motivations of each reformer.”

•What is a “Loose hypotheses? Is this scientific?

“While COPUS data could clearly differentiate levels of reform, the

•attitudes discovered in the interviews was [were] not able to clearly differentiate between reformer types. However, the identified themes can offer insight into how attitudes, subjective norms, and perceived behavioral controls can influence desires to reform.”

“Future professional development programs should understand that in the case of

both advanced and student-centered reformers, faculty had to deal with mixed feedback from

students. Their chosen degree of student-centeredness may depend on how committed they are to the philosophy of SCT.”

•Again, the use of “understand” is problematic because the meanings of the term are so broad -- perhaps replace with “consider”

•p.8 The STEMFI program lasted three to four semesters (over two years) and consisted of three

phases: pre-, during, and post-workshop. Does this mean for some participants it was 3 semesters, others 4 semesters? The duration of the program was 4 semesters plus summers, is that correct?

•We tried to pair mentors and participants who were in the same discipline •delete~~(so that they understood the disciplinary nuances)~~ but outside of the department (so that they had no influence on rank or status decisions).

•p.10 Participants were made aware of the observation •delete~~, ‘but the observer was usually a student who blended in with the class.~~  Faculty would recognize a new person, right? Is “blending in” an appropriate statement?

Most participants in the program displayed “fully reformed” attitudes, especially among those who were primarily didactic in their approach to teaching (i.e., beginning and dramatic reformers).

•Question the use of “fully” in this context. Do the data support “fully”?

Discussion -- Overall, the discussion section needs reduction in length and careful editing (pay particular attention to noun and verb ‘number’ agreement). I recommend creating a Table that highlights the characteristics of each category used in Figure 2. Easy reference for the text.

•p. 26 “If a shift to SCT is intended to give students more control over their learning experience, departments may need to reevaluate their expectations for content coverage in favor of deeper learning of fewer topics.”

•The big idea here is curriculum. Recommend highlighting “curriculum” as the major issue.

Figure 1 - unnecessary

Figure 2 - nicely done and helpful

Conclusions:

“According to the established literature, students who learn through a more student-centered

approach have a greater overall retention of information [e.g., 31].”

•This statement is based on the reader’s definition/conception of “learning” and is subject to debate. Is learning retention of information? I recommend deleting this sentence and beginning with what the data show in this study.

I think the authors can claim that teaching practices are malleable based on the data presented.

•Use of COPUS as the observation tool is limiting.

However, the use of SCT is interesting and novel in the context of this research.

•A major question that I encourage the authors to consider in the future is this: After (and even before) STEMFI what did the assessments in the target courses look like? Were students asked to repeat information? Or were students asked to do something with the knowledge/core concepts using science practices? Each participant’s definition of SCT should affect the assessments of student learning. Basically, we can change teaching, as described here, but if the assessments (of student learning) don’t change, what have our students gained?

I enjoyed reviewing this paper.
