# Supplementary material for: Iterating toward change: Improving student-centered teaching through the STEM faculty institute (STEMFI)
Source: PLoS One. 2023 Aug 17;18(8):e0289464. doi: 10.1371/journal.pone.0289464 (PMC10434963; doi:10.1371/journal.pone.0289464)
Supplement: Attachment — Submitted filename: Response to Reviewers.docx [file pone.0289464.s007.docx]

Response to Reviewers

Reviewer #1: May 6, 2023
Re-Review PLoS One PONE-D-22-33194

The authors have done a very nice job of revising their manuscript based on my initial comments. I find the manuscript is now easier to read and presents a stronger case for their findings. I have only a few minor changes and I do not need to rereview these minor changes.

Unfortunately the manuscript did not come with line numbers so I will only be able to use page number to indicate areas for change.

Page 7
You start off this section indicating that the program consisted of three phases: pre-, during, and post-workshop. You indicate the Pre-workshop content and in paragraph 2 on page 8 you identify phase three. Where is Phase 2. It would be nice if paragraph one on page 8 began with the phrase, Phase 2 involved faculty participation in a on-time workshop.
On the other hand you could just drop the use of the term “phases:.

Good suggestion. We have added this phrase for clarification.

Page 9 top
“observed using COPUS to measure their post teaching behaviors. Participants chose which classes would be observed.”

Please remove the sentence “Participants chose which classes would be observed” as you more fully explain this in the next section about Observations. This much detail feels out of place here.

Good suggestion. We have removed this sentence for clarification.

Page 9 bottom
“Post-observations were made in the first semester they taught following their participation and were requested by the participant in order to showcase the new techniques they were planning to use.”

This is a nice sentence that addresses the non-random selection of post-workshop courses to COPUS.
The word “selected” would be a better word than “requested”

Thank you for this suggestion. We have adjusted the wording.

Page 10
“likewise those who were already using student-centered strategies and continued
being student-centered were labeled “Student-Centered Reformers”.
I found it odd that a faculty member that started as student-Centered and remained at student -centered would be titles a “reformer” as in fact they did not reform they just kept doing what they were already doing. If however they “broadened their strategies’ as was stated for the interactive to interactive faculty than I could accept labeling them as reformers. Please add more text here to show how they can be considered “reformers”.
This will also impact your statement on page 12 that states 35 of 41 participants changed their teaching enough to be “reformers”. If faculty who remained in their category ( Inter to Inter, or SC to SC) and did not add new strategies, they should not be termed reformers.

We have added more text to page 10 and 12 to clarify the description of classification.

Page 13-top of page.
“so they can only hint at potential differences between reforming attitudes.”

I would rewrite as “so they can only hint at potential differences between reformers.”

Thank you, we adjusted this wording.

Page 13
You italicized the term in transition but did not italicize the dichotomus pair “Fully Reformed”. As you do not italicize the dichotomous pairs under the other themes please remove the italics from in transition.

Adjusted.

Page 21
Student-centered reformers were those who already had demonstrable experience implementing SCT strategies coming into this experience. Their attitudes can teach us several
things. First, we see fully reformed attitudes among this group

The middle sentence, Their attitudes.. needs a rewrite. I suggest the following
Student-centered reformers were those who already had demonstrable experience implementing SCT strategies coming into this experience. We found that this groups has fully reformed attitudes.

Great suggestion. We have implemented the edit.

Page 21- bottom
Non-reformers displayed many of the same attitudes as those who chose to make
measurable changes. However, we can learn a few things from them that can help inform future efforts.
The second sentence felt a bit too casual with your reader. I would just remove it.

We have removed colloquial wording.

Page 24 Conclusions
“According to the established literature, students who learn through a more student-centered
approach have a greater overall retention of information [e.g., 31].”

I have read citation 31 Smith et al. 2014. They conducted a survey of teaching practices across their university using COPUS and had faculty self-report the practices they use. They noted a high degree of alignment between COPUS results and self-reports. I did not see any mention of “greater overall retention of information”. Please remove this citation.
You may need to remove this whole last sentence because I am unaware of any established literature that has shown greater overall retention of information. There are many citations that support increased exam scores but none that I know of that deal with retention.

This is an excellent point. We have removed the sentence entirely.

Reviewer #2: Overall, my biggest feedback is what Reviewer 1 previously mentioned about the post-observation not being at random and only occurring the single time when the pre-observation occurred multiple times. This is problematic as the instructor could be performative in their methods and not actually adopting SCT strategies. Additionally, only doing the post-observation once, is a similar issue that leads for the data to not be as generalizable on if the class was a one-off or if the instructor has really adopted SCT strategies. Overall, I think to help remediate this, the categorization of faculty needs to be written differently as you can’t confirm that they are now student-centered instructors, but rather that they can use the techniques efficiently. This needs to be made clear in the manuscript.

Throughout the manuscript we have added and softened the claims that the participants became student-centered instructors, but rather that they can use the techniques now more efficiently.

Reviewer #3: I appreciate the edits made to further clarify the results and methods sections of this manuscript. The faculty professional development community will greatly benefit from the review of this study and program.

Thank you for your time in reviewing and encouragement. We are excited to share our results.

Reviewer #4: This submission is interesting, well written and an important topic. The authors discuss the theoretical concepts leading to their intervention, which adds to the depth of the submission. The intervention and it's evaluation are also multi-layered and the authors have already made some helpful changes to assist the reader in understanding their results e.g. Figure 2. I would like to request some minor changes.

1. Please describe acronyms fully when first introduced. The paper assumes prior knowledge e.g. STEM. COPUS is described but only on Page 7 when the acronym was introduced much earlier.

We have clarified the acronyms. Thank you for pointing out this edit.

2. The link provided to the data repository didn't list the study <https://scholarsarchive.byu.edu/> - please provide full details about where the data is located.

Thank you for the reminder. We were waiting to post it, until the manuscript was accepted. We have fixed the URL to access the data that is now posted: https://scholarsarchive.byu.edu/data/49

3. The authors discuss the concept in the introduction that "development programs frequently do not cause lasting changes to teaching strategies or student engagement" - in the discussion please address how you plan to overcome this issue. I wouldn't classify the current duration of observation as 'long-term'.

In the discussion we have qualified our perspectives on lasting change based on the evidence from this study. Thank you for helping us connect these ideas in our paper.

Reviewer #5: This study evaluated the effectiveness of a STEMFI on student-centered teaching. Both classroom observations with COPUS and post-interviews were used for evaluation. The results showed that faculty shifted toward student-centered teaching after the STEMFI program. The manuscript is well-written, and the findings are interesting. However, some important clarifications about the results are needed. 1. COPUS captures teacher and student behaviors (e.g., lecturing, asking questions, group work), but it does not capture the specific student-centered strategies (e.g., think-pair-share, 5E learning cycle). Faculty shifted toward student-centered teaching could be because they spent more time on group work, but does not necessarily mean they used more student-centered strategies. Also, the characteristics of each COPUS profile (in terms of the COPUS codes) need to be discussed to help readers interpret the results, especially those who are not familiar with COPUS. Please clarify for each type of reformer, what student-centered strategies they tried after participation, or they used the same strategies as before but spent more class time on those strategies. 2. I am concerned about the reliability of the observation data. How were graduate and undergraduate students trained for using COPUS? Have you investigated the inter-rater reliability?

We have responded to each of the detailed comments below.

More detailed comments below:
1. In my opinion, COPUS codes and the characteristics of each COPUS profile should be discussed in more detail in order to help readers interpret the results. COPUS codes are somewhat general, which doesn’t capture specific student-centered strategies. I think the authors need to be more carefully describing how faculty reformed their teaching. Did they spend less time lecturing but more time on group work, or did they implement some new student-centered strategies, such as 5E learning cycle and POGIL? Also, I think you also need to give more details about the strategies introduced in STEMFI. For example, what is POGIL if someone doesn’t already know.

The COPUS measurement allows us to distinguish active learning strategies into a few categories. Codes include more teacher-centered approaches, such as lecturing by the instructor and listening by the students, more interactive strategies like student questions and instructor answers, and more fully student-centered strategies like group work, clicker questions, and the instructor moving and guiding around the room. It does not, however, assess specific active techniques. For more description of the instrument and codes, see Smith et al 2018. We have clarified the manuscript to include this description. Thank you for pointing out this edit. Additionally, we have included brief detail of the workshop but referred readers to our other publication where the workshop is described in more detail.

We have added information and an additional citation about the specific strategies introduced in STEMFI.

2. It is unclear to me how graduate and undergraduate students were trained for using COPUS. How many students did the observations? How were they assigned for observations for different faculty? What is the inter-rater reliability?

We used the training protocol outlined in Smith et al. This is already included in the manuscript. This is a common training protocol used for the COPUS. (“ Undergraduate and graduate students were trained to use the COPUS using the training protocol established in Smith et al. [21].”)

3. On page 12, the authors said some post-data was collected on hybrid or online courses. How did it affect the classroom observations? I imagine it is hard to do COPUS with online courses. Also, how did it affect the COPUS profile? Were online courses more likely to be didactic?

We have added a clarifying statement that addresses the data collected on hybrid courses and how it affected the classroom observations.

4. Thematic analysis. How many researchers coded the transcripts? Did you always have two researchers do independent coding first?

Please see the description that was already revised from previous reviewers under the heading “**Interview protocol for qualitative analysis”**

5. Please give more details about the triangulation of COPUS and interview results. Please say more about the explanatory mixed methods design, and how you “merge” the findings.

Previous reviewers suggested that the triangulation was not clear. We significantly re-wrote the qualitative portion, responding to reviewers as such:

“This is an interesting perspective and it made us re-think how we described our categories. We agree that it was difficult to differentiate among the reformer types with the qualitative data and we decided that perhaps that was not the point. We have gone in and rewritten the qualitative section to be more about the four themes that emerged in conjunction with the TPB framework, with suggestions for trends that match the profiles. But, we emphasize that it is not always a direct relationship between them (i.e., there is a lot of overlap). We have also included descriptions of our didactic non-reformers.”

We also added to our manuscript, the following: “Through the lens of the Theory of Planned Behavior, we triangulated these categories with COPUS data to make loose hypotheses about the motivations of each reformer. However, we found that the themes were not robustly tied to specific reformer types in an exclusive way, so they can only hint at potential differences between reformers.”

Without more details about what you’d like to see, it is difficult to know what additional information is needed.

6. Did the faculty need to apply to get into the program? If so, how many applications each year? Also, I think those who applied are the ones more interested in SCT, and may have more positive attitudes toward SCT. Did you do pre-interview? Any chance those participants already had positive attitudes toward SCT before the program? Can you please comment on how this can affect your findings?

Thank you for this suggestion. We have included a clarification and future research direction in our limitation section about our sample that may have already had an increased interest in reforming their instruction.

7. You have student responses and challenges as two separate themes. The literature has shown that students’ negative responses is one barrier for reformed teaching. Can you please comment on why you decided not to include this as a challenge? On page 18, there is a quote of student negative feedback as a perceived challenge.

We recognize that negative student responses are a challenge (we added an additional sentence to clarify), but we separated this as a different category because there were also positive student responses. This is an important idea, however and we see this as a future avenue for specific and directed research. We have added a clarifying statement in the manuscript.

8. The development of the interview protocol was informed by TPB. Am I understanding it correctly that the students were considered “significant others” and students’ responses gave subject norms? If you could be more explicitly on how each of the three factors informed the interview questions, that would be great.

Thank you for this clarifying point. Yes, you are correct, students were considered the “significant others” that were providing feedback. But, we also considered peers and administration in our interviews. We have added details under the interview protocol for qualitative analysis to demonstrate how we developed the interview questions based on TPB.

9. You included faculty ranking when reporting the results. Did you see any patterns of faculty at different rankings?

In this iteration we didn’t have sufficient participation at each rank to see any meaningful patterns. Additionally, rank data was added at the request of a previous reviewer. We agree that this would be an important idea to flush out in future research questions.

Thank you for your work! Looking forward to your responses.

Reviewer #6: An excellent addition to the literature on faculty teaching development. The authors have responded well to the comments from initial review and made updates that improve the quality and readability of their manuscript. I have no further feedback to offer, and look forward to seeing this manuscript published!

Thank you for the feedback.

Reviewer #7: There are minor revisions (e.g., use of terminology, condensing the Discussion). Careful edit of the text is necessary.

We are unable to address the concerns of this reviewer since no details were offered. We have read through the manuscript to look for any grammatical errors and fixed any we found.

Reviewer #8: Summary:
This study attempts to examine the impacts of a faculty professional development program, STEMFI, on the impacts on instructional practices (COPUS data) and instructor’s attitudes, perceptions of students’ responses, and confidence (interview data). The study appears to be well aligned to the selected theoretical framework, The Theory of Planned Behavior. And there are some clear visual representations in Figures 1 and 2. Unfortunately, there are quite a few major revisions that need to be addressed before this manuscript is considered any further: (1) focus is on examining student-centered teaching (SCT), when I think it should be on active learning; (2) educational problem(s) and/or research question(s) are absent (or at least unclear); (3) COPUS data are presented in a qualitative manner, but authors claim that these are quantitative results; and (4) mixed methods research design is unclear. I suggest that the authors rework the introduction and methods with the suggestions below in mind.

Major issues:
Freeman et al. (2014) meta-analysis focuses on the impacts of active learning on student performance outcomes, not student-centered teaching (SCT). I would be careful to not conflate active learning and SCT. I would suggest that the terminology is changed from SCT to active learning since not all active learning is necessarily student-centered.

We have clarified in the manuscript on the STEMFI focus upon student-centered strategies. And we have modified the manuscript in places where we are just referring to active learning and places where we are specifically focused on SCT. We do not want to rename SCT throughout because our STEMFI program was specifically focused on SCT.

Relatedly, is there a reason that you decided to write your own definition of SCT (or what I am suggesting is active learning in my point above)? Why did you not want to use a definition from the literature? I suggest that you review this CBE LSE paper on active learning for potential literature-based definitions: Driessen, E. P., Knight, J. K., Smith, M. K., & Ballen, C. J. (2020). Demystifying the meaning of active learning in postsecondary biology education. CBE—Life Sciences Education, 19(4), ar52.

Reviewer #1 in the original response to reviews brought this up and we decided that student-centered teaching was the better term to use. See our response: We prefer the term “Student-Centered Teaching” to emphasize the constructivist approach that we were teaching. Active learning seems too broad (i.e., teaching can be active and not constructivist). Evidence-based practices is also just not as descriptive. We have made sure we consistently use that term throughout.

The educational problem(s) and/or research question(s) are not clearly stated in the abstract nor at the end of the introduction. I am not sure what is being examined in this study, so it will be hard for me to determine if the methods are appropriate and well-aligned with the educational problem and/or research question.

We have clarified our research question at the end of the introduction.

While Figure 2 is clear for understanding how groups of instructors shifted from one COPUS profile to another, I don’t think that Figure 2 is sufficient for the reader to be able to understand the quantitative nature of the COPUS results. Right now, the results are described in a qualitative manner, which is okay; however, the authors claim that the results are quantitative. I would suggest that you give an example of the instructor and student behaviors occurring in each of those six types of reformer classrooms. See Shi et al. (2023) CBE LSE (<https://doi.org/10.1187/cbe.22-03-0047>) for an example of figures.

We have cited Stains et al. in our methodology of classification. We have further described our classification of reformers based on the instructor and student behavior data. Additionally, we feel like Figure 2 is representative according to previous reviewer comments by reviewer #1: “The categories you created to classify participants are very clever and the figure 2 is a nice representation of that categorization method.”

Also, the mixed methods research design is unclear to me. Right now, it appears that COPUS data were collected, and interview data were collected, but how one dataset might inform the other needs to be further explored by the authors to answer the research question(s).

Please see our comment above.

Minor issues:
Introduction:
Page 3
Please add a reference to the last statement on this page about the primary objective of these workshop. In addition, I think that “knowledge” is missing from the list of primary outcomes of faculty professional development programs.

We removed this sentence. It seemed redundant. We agree that knowledge should be a desired outcome, but we did not find studies that measured this.

Methods:
Page 7
Participants: Did you collect any other demographics data from the instructors? Or only discipline and gender (in the binary)? Also, could you please describe the student population being served by these instructors being studied? And finally, please describe the process for recruiting these instructors.

No, we did not collect any additional demographics on these instructors. We also did not collect demographics from their students. However, their students would presumably be representative of the student body at the host institution , details of which we have now included under “Participants” in the methods section.

Page 9
How did you ensure that the students were trained to code COPUS in a reliable manner? Did you calculate inter-rater reliability after this training? Please provide more details on how you tested for coder reliability.

(Please see our comment above - we used the Copus training program outlined by the authors of the instrument. We did not systematically collect IRR, but we followed the protocol until we got to agreement.)

Page 10
I was not able to see the supplemental information to be able to see the full interview protocol. Also, it would be helpful if the supplemental file information (e.g., Supplemental File S1) was noted in the text.

Thank you for noting this missing file. We will include this file in the final submission. And we have indicated it in the text.

Page 11
Who are the several researchers that thematically coded the interview responses? I think it is important to be transparent about which specific researchers did this work to build trustworthiness in the data.

We have changed the wording in the manuscript to indicate which researchers thematically coded the interview responses.

How exactly did you connect the quantitative and qualitative data using exploratory mixed methods design? It is unclear to me which COPUS and which interview data were used for these analyses. It is not until the results that I read that themes were not tied to specific reformer types. It is important that you bring up these details in the methods, not just results.

This is an excellent point. We have duplicated the statement from the results in the methods section.

Results:
Page 11
I would move this participant info to the methods section. It’s good to be transparent about the loss of participants due to COVID, but I don’t think it is a great way to start your results section.

We have adjusted the participant info in the results section.

Why did you decide to add the ranking of the instructors to the quantitative results? Are you interested in examining how instructor rankings impacted use of student-centered teaching practices? Again, I am not sure if these results are aligned to the research questions and methods as I did not clearly see research questions earlier on.

We only added this in response to a previous reviewer who requested it. We were not interested in analyzing reform by rank as we did not have enough participants in each category to really identify any meaningful patterns.

References:
Check first author spelling for this citation: Creswell, J. W., & Plano Clark, V. L. (2007). Designing and conducting mixed methods research. Thousand Oaks, CA: Sage.

We fixed it! Thank you for catching this!
